# Enhancing Postoperative Virtual Visits in Vascular Surgery: A Quality Improvement Initiative Amidst COVID-19

**DOI:** 10.7759/cureus.82156

**Published:** 2025-04-12

**Authors:** Mini George, Bushra M Manakatt

**Affiliations:** 1 Vascular Surgery, MD Anderson Cancer Center, Houston, USA; 2 School of Nursing, University of Texas Medical Branch, Galveston, USA

**Keywords:** covid-19, discharge education, elderly patients, nursing care, patient education, postoperative virtual visit, staff training, vascular surgery, virtual assessment, virtual care

## Abstract

Introduction: In the realm of healthcare, virtual care has emerged as a pivotal tool for patient-provider communication, particularly amid the challenges posed by the COVID-19 pandemic. In vascular surgery, the adoption of postoperative virtual visits has proved to be a safe, cost-effective, and efficient means of monitoring patient recovery while minimizing the need for in-person clinic appointments. But despite its potential benefits, the use of virtual postoperative visits remains suboptimal, prompting the creation of a quality improvement initiative aimed at enhancing their use.

Background and local problem: Virtual postoperative visits play a crucial role in timely identification of complications such as wound infection, hematoma, or graft failure in vascular surgery patients. Yet even as the pandemic necessitated a shift towards virtual care, the rate of postoperative virtual visits in a cancer center remained steady at around 30%. Recognizing this as a local problem, efforts were directed towards increasing the utilization of virtual postoperative visits through targeted interventions.

Methods and interventions: The quality improvement initiative encompassed several key interventions within the discharge process: a) implementation of scheduled virtual visits post-education and patient discussions pre-discharge; b) modification of the electronic health record to include smart phrases facilitating virtual visit education and built-in reminders for follow-up scheduling; c) adaptation of patient education materials to incorporate real-time demonstrations and keywords for virtual assessments; and d) staff training on virtual evaluation techniques for incisions and postoperative complications.

Results: Following the implementation of these interventions and iterative Plan-Do-Study-Act (PDSA) cycles, the rate of postoperative virtual visits surged to over 50% and was sustained. A sample of 49 patients revealed an impressive 87% completion rate for virtual visits within three weeks post-surgery. Additionally, associated benefits included a reduction in clinic no-show rates, 30-day readmission rates, and surgical site infections. Notably, the teach-back method emerged as an effective strategy for educating elderly patients on virtual visit technologies, underscoring the feasibility of virtual postoperative care among this demographic.

Conclusion: The successful enhancement of postoperative virtual visits in vascular surgery not only underscores the safety and feasibility of virtual care but also offers avenues for future research. From exploring the impact of nursing care and patient education to ensuring equitable access among older adults, virtual postoperative care stands as a promising frontier in enhancing patient outcomes and optimizing healthcare delivery.

## Introduction

Vascular surgery patients with comorbidities such as cancer, diabetes, lung diseases, obesity, and decreased functional status have a 25% readmission rate within 30 days after surgery [1,2]. Providing appropriate discharge instructions and scheduling follow-up visits within three to four weeks after surgery can reduce the readmission risk by enabling the timely detection of complications such as wound infection, hematoma, or graft failure [1,3,4]. The recent pandemic led to a reduction in elective vascular surgeries and the replacement of traditional postoperative follow-ups with virtual care [5]. Virtual postoperative visits have proven to be safe, cost-effective, and beneficial in increasing both patient and provider satisfaction [6-10].

Patient reluctance and lack of confidence in virtual care limit the adoption of virtual visits [11-13]. Providing a clear explanation and demonstration of the virtual visit experience can help make patients more comfortable with accessing virtual care [14,15]. Educating patients about virtual care can make virtual visits easier and more effective, eliminating transportation issues and enhancing overall care [6-8,11].

Background

The pilot project was conducted in the vascular clinic at a tertiary cancer hospital in Texas. This clinic typically schedules postoperative visits within the recommended time frame, which expert opinion suggests should be within four weeks after vascular surgery [1,3]. In response to pandemic preparedness in March 2020, the vascular clinic replaced traditional postoperative visits with virtual visits via the web portal ‘MyChart.’ However, the rate of timely postoperative virtual visits dropped to below 30% due to patient refusal, the unavailability of face-to-face visits, technological difficulties, and other challenges associated with the new program.

Initially, postoperative discharge education did not include information about virtual visit scheduling, navigation, or expectations. After discharge, clinic staff provided information about postoperative virtual visit schedules and navigation via telephone or electronic messages. The lack of pre-discharge education on virtual visits made the process challenging for both patients and staff, and patient reluctance to schedule virtual visits further decreased their occurrence. This situation likely contributed to multiple issues, such as delays in postoperative medication adjustments, complications with surgical wounds, and disruptions in adjunct cancer therapies. Consequently, the delayed virtual postoperative visits led to dissatisfaction among both patients and employees.

Experts have recommended continuing non-emergency cardiovascular care virtually during the pandemic [5]. Virtual postoperative care can minimize viral exposure, provide excellent clinical outcomes, and increase patient satisfaction while saving costs for both patients and the healthcare system [16-19]. Several factors influence virtual care, including patient selection, patient engagement, provider-patient relationships, training, technology, reimbursement, licensing, liability, data integration, privacy, and security features [11,19-23]. Initially pioneered by NASA for the live monitoring of astronauts' health [12], virtual care or telemedicine has since been adopted by many institutions. Various modalities are used, including SMS text messaging, smartphone applications, automated telephone calls, wearable devices, and cloud applications using electronic health records [12-14].

Postoperative management requires strict adherence to follow-up schedules. Vascular surgery patients are seen within one month of discharge for wound and vascular assessments [1,4]. Common postoperative complications and top reasons for 30-day readmissions after vascular surgery include wound-related issues and surgical site infections [1,3]. Patients who have undergone lower extremity revascularizations, are non-independent, or have conditions like low hematocrit, chronic obstructive pulmonary disease (COPD), or renal issues, have higher rates of ER visits and readmissions [2,3].

Virtual care can safely transition surgical care during the postoperative period by saving time, expenses, and travel for patients [12,18,20]. Studies indicate that provider management and 30-day readmissions for wound care and postoperative complications do not differ statistically between virtual and traditional postoperative care [6,9,20,21]. Digital images sent by patients or caregivers are effective for accurate postoperative wound care assessment and early identification of complications by providers [11,20,24].

## Materials and methods

Study design

This prospective, observational study aimed to assess the impact of discharge process modifications on increasing the rate of virtual postoperative visits in a vascular surgery clinic. The intervention involved changes to the discharge process, including patient education, scheduling of virtual visits before discharge, and modifications to the Electronic Health Record (EHR) system. Data were collected over a four-month period, from August to November 2020, to evaluate the effectiveness of these changes in promoting postoperative virtual care.

**Figure 1:**
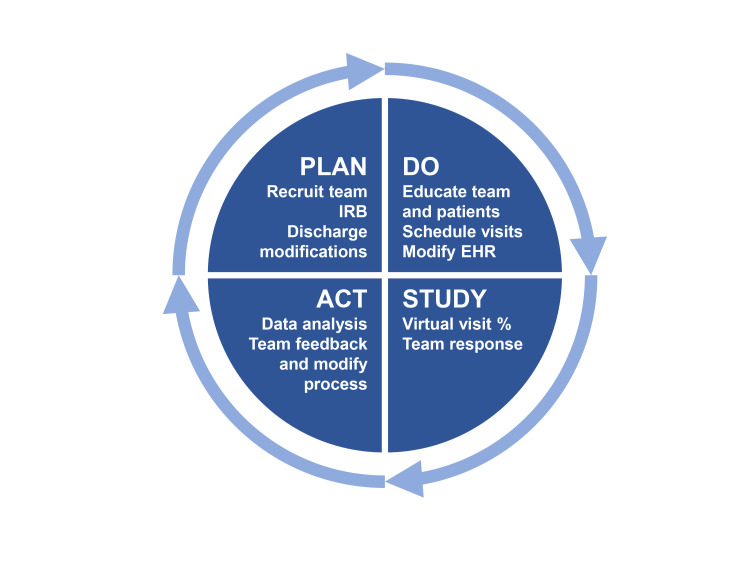
Figure showing Plan-Do-Study-Act (PDSA) cycles

The study utilized the Model for Improvement framework, which guided the development and implementation of process changes. The project employed the Plan-Do-Study-Act (PDSA) cycle methodology for continuous testing, adjustment, and evaluation of the intervention (Figure 1). Outcomes were measured by the change in the rate of virtual postoperative visits, adherence to discharge process modifications, and stakeholder feedback.

Study population and sample size

The study population included adult cancer patients undergoing complex vascular reconstructive surgeries at a tertiary academic cancer center in Texas. The inpatient unit accommodates 15 adult patients and maintains a 3:1 patient-to-registered nurse (RN) ratio. The unit is supported by a multidisciplinary team, including designated supportive staff, a physical therapist, a pharmacist, a social worker, and an advanced practice provider. At the hospital, the electronic patient portal includes Health Insurance Portability and Accountability Act (HIPAA)-compliant videos for virtual follow-up visits. To access these virtual visits, patients need to install video-capable applications on their smartphones, tablets, or computers. However, 50% of vascular surgery patients over 70 years of age have expressed concerns about using these new technologies, as well as about maintaining proper communication and continuity of care after discharge. Consequently, many of these patients preferred to opt for regular, in-person postoperative clinic visits instead.

The sample size was determined by the total number of patients undergoing postoperative care during the study period. Data were collected from the EHR system, which included records for all patients who had undergone surgery during the study window. The sample size was based on convenience, as the entire cohort of patients who met the eligibility criteria was included in the analysis. While a specific power calculation was not conducted, the data were expected to be sufficient for trend analysis due to the consistent volume of surgeries at the center.

Study measures

Primary Outcome

The primary outcome measure was the rate of virtual postoperative visits, defined as the proportion of patients who completed a documented virtual follow-up visit with a healthcare provider post-discharge. This was compared to baseline data from the clinic's prior virtual visit rate (30%) before the project began.

Secondary Outcomes

Documentation of discharge education: Whether patients received and understood virtual visit instructions as part of their discharge education. This was recorded as a yes/no indicator for each patient.

Scheduled virtual visits: The proportion of patients with scheduled virtual visits before discharge, compared to those who were scheduled for in-person visits.

Patient satisfaction and feedback: Collected via institutional surveys distributed to patients and stakeholders (including healthcare providers and staff). This qualitative data provided insight into the perceived effectiveness of the intervention.

Adherence to modified discharge process: This was assessed by measuring the completion of key discharge process components, including virtual visit education, scheduling of virtual visits before discharge, and use of the After-Visit Summary (AVS) document.

Ethics Statement

The study was conducted in compliance with ethical standards set forth by the Institutional Review Boards (IRBs) of both the hospital and university. Informed consent was obtained from all participants, ensuring that they were aware of their involvement in the study and the purpose of data collection. The study adhered to HIPAA regulations to maintain the confidentiality of patient data, which was stored in a secure, password-protected Excel spreadsheet (Microsoft, Redmond, WA, USA) hosted on institutional cloud storage.

The project was reviewed and approved by the IRBs prior to the commencement of data collection. The IRBs also reviewed the study for potential risks to participants and determined that no significant risks were involved, as the study primarily involved retrospective data analysis and the review of routine clinical practices.

Data collection

Data were collected through the EHR system, where records of all patients undergoing postoperative care during the study period were reviewed. 

Key Data Points Extracted From the EHR

Discharge education documentation: Whether patients received virtual care instructions and if these instructions were linked to the AVS document.

Scheduled virtual visits: The presence of scheduled virtual follow-up visits recorded in the patient’s discharge summary.

Completed virtual visits: Confirmation that the patient completed the virtual visit, as documented in the EHR.

Demographics: Patient age, type of surgery, and other relevant demographic information were recorded for further analysis.

Data were securely stored in the institutional cloud storage system, ensuring compliance with privacy regulations.

Statistical analysis

Statistical analysis was performed using descriptive statistics to summarize baseline characteristics and the outcomes of interest. The primary analysis involved comparing the pre- and post-intervention rates of virtual postoperative visits. The percentage increase in virtual visits was calculated, and statistical significance was assessed using appropriate tests (e.g., chi-square or t-tests) to determine whether the intervention significantly affected the virtual visit rate.

Additionally, descriptive analysis was performed on patient satisfaction data collected from institutional surveys to assess stakeholder perceptions of the discharge process modifications and virtual care instructions.

Subgroup analysis was conducted for patients over 70 years of age to determine if the modifications had a specific impact on this population, given their initial concerns about technology use. This group was analyzed separately to assess if the interventions successfully addressed their specific barriers to virtual care adoption.

Stakeholder feedback and qualitative data

Stakeholder feedback was gathered through surveys distributed to healthcare providers, nurses, and other clinical staff involved in the discharge process. These surveys aimed to assess their perceptions of the modified discharge process, any challenges encountered in implementing the intervention, and suggestions for further improvements. Feedback from patients was also collected to understand their experience with the virtual visits, including ease of access, clarity of instructions, and overall satisfaction with virtual follow-up care.

Qualitative data were coded and analyzed for recurring themes related to the effectiveness of the discharge process modifications and any remaining barriers to virtual visit adoption.

## Results

**Figure 2:**
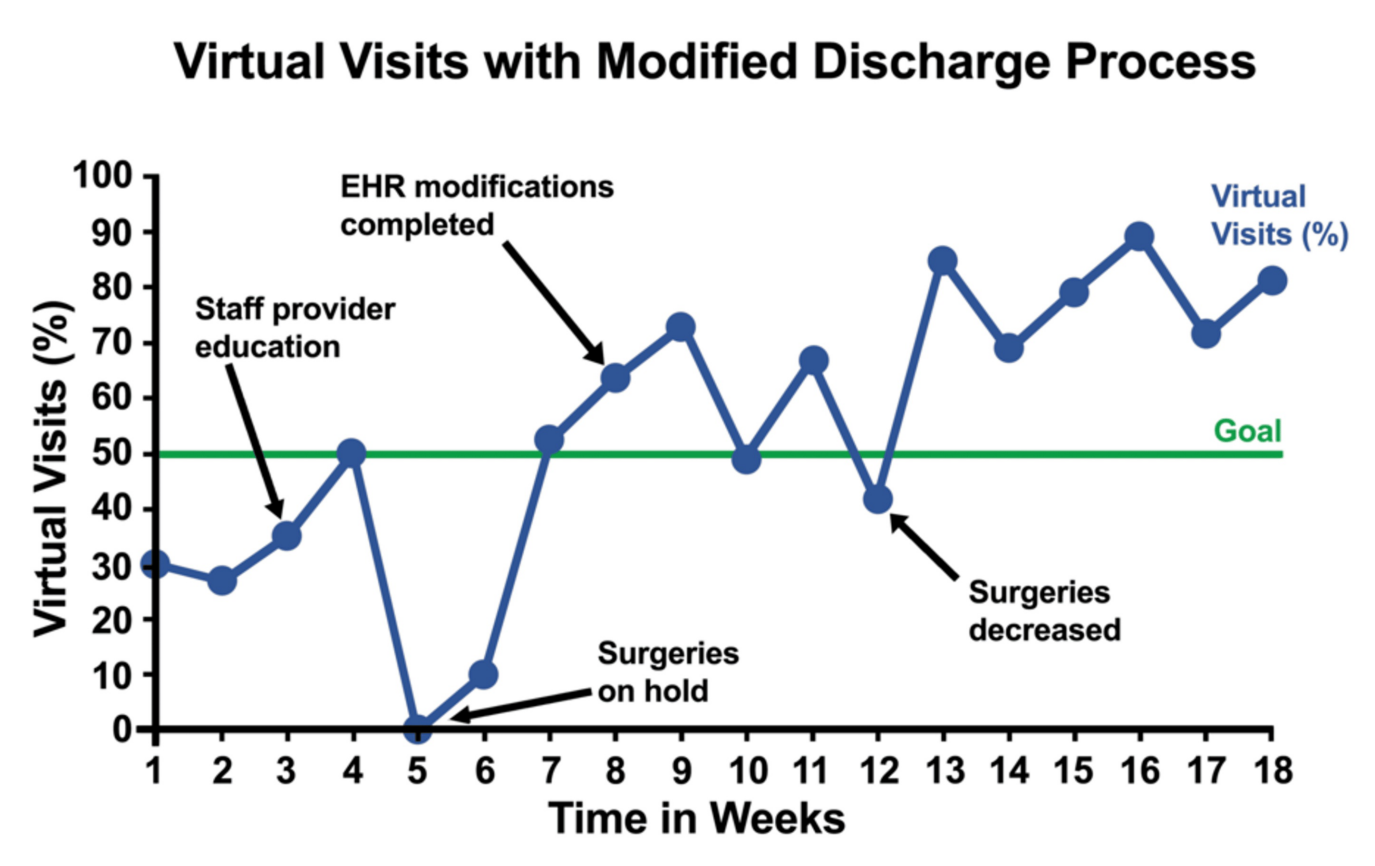
Run Chart showing Virtual Visits Rate After Discharge Process Modifications EHR: Electronic Health Record

During the project, the virtual visit rate fluctuated from the goal even after implementing all discharge modifications. The run chart (Figure 2) showed that the visit rates had a random distribution of 10 runs. The chart recorded no positive or negative trend at the beginning. By the end of the project, the virtual visit rate remained above the goal and the visit rates started to have a positive trend.

The run chart also compared the outcome measure from weekly analysis of virtual visit rate (Figure 2). The x-axis and y-axis of the run chart represented the time in weeks and the virtual visits rate. The baseline rate was 30%. The target goal was to increase virtual visit rates to 50%.

From the sample of 49 patients, the EHR was reviewed to assess the documentation of all relevant process measures. These process measures included the following: (1) Percentage of patients receiving virtual visit discharge education. This measured whether patients were provided with educational materials and instructions about virtual visits as part of their discharge process; (2) Percentage of patients with a scheduled virtual visit before discharge. This assessed whether virtual follow-up visits were scheduled with patients prior to their discharge from the hospital; (3) Percentage of completed scheduled virtual visits. This tracked the completion of virtual follow-up visits, confirming whether the scheduled virtual visits were actually conducted after discharge.

These measures were used to evaluate the adherence to the modified discharge process and the effectiveness of the interventions aimed at increasing virtual postoperative visits.

**Figure 3:**
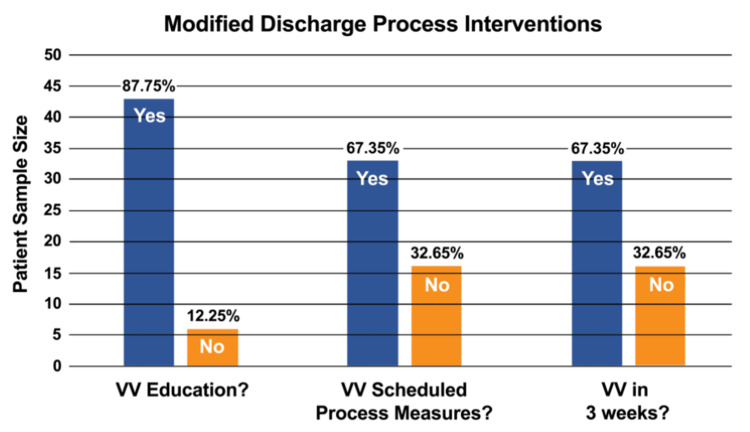
Column Chart of Proportion of Completed Discharge Process Interventions VV: virtual visit

The project leader reviewed the patients' charts for implemented process measures such as virtual visit education, virtual visit schedule before discharge, and completion of virtual visits within three weeks of surgery. The blue column in Figure 3 represented those patients who completed process measures, and the yellow column represented those who did not do the process measures. As shown in Figure 3, 87.76% of patients had virtual visit navigation education before discharge. 12.24% of patients who missed explanations and demonstrations of virtual visit navigation were those who refused education or those who were discharged during a low staff-patient ratio or within six hours after surgery. 87.76% of all patients received virtual visit education, and 67.35% were scheduled and completed virtual visits. The difference of 20.41% was the attrition rate of virtual visits during the project. The difference of 20.41% was the attrition rate of virtual visits during the project. Ten patients did not complete visits, and the others were not scheduled for visits during the weekends.

89.94% of the total staff attended one of the three virtual educational sessions about discharge process modifications. Four of the employees who were absent for classes were on prolonged leave. The educational materials are stored electronically on the department's shared drive for ease of reference. The run chart showed a positive shift after the staff education sessions, indicating increased staff adherence to discharge education.

From August to November 2019, 30.77% of total clinic visits were regular or traditional face-to-face postoperative visits. During the project timeline of August to November 2020, only 8.59% of traditional postoperative visits occurred. Therefore, the 22% of postoperative clinic visit slots saved during the project can lead to a potential increase of allotted time for other types of clinic visits. During the project, two natural disasters and a global pandemic decreased the timely scheduling of surgeries and follow-up visits. Hence, the reduced number of elective surgeries, schedule conflicts, and patients' refusal for virtual visits became the unmitigated process deficiencies. Missing data included two patients who had surgeries during the first weekends of the project and a patient discharged for home hospice.

After virtual care initiation, postoperative virtual visit scheduling became a cumbersome effort in our clinic. During the practice project, the multiple discharge process modifications resulted in a substantial increase in safe, timely, efficient, and patient-centered postoperative virtual visits. The teach-back method of virtual visit navigation demonstration with a conveniently scheduled virtual visit increased patients' adherence to postoperative virtual visits, reduced clinic no-shows, and schedule adjustments. Another relevant outcome of the project is the successful application of cost-effective health care technology for postoperative care among older adults. 

## Discussion

This process improvement project aimed to increase the postoperative virtual visit attendance rate to 50% in the vascular clinic by targeted interventions. Modified discharge process with patient education, EHR modifications, and staff education during the project were detrimental in sustaining the timely postoperative virtual visit rate above 70%. The project identified a clear association between the postoperative virtual visits within three weeks after discharge and the postoperative virtual visits scheduled based on the patient’s feedback during hospitalization. Another significant outcome of increased virtual visits was the 22% reduction in traditional postoperative clinic visits with revenue recovery.

There was no unplanned 30-day readmission related to vascular surgery among the 49 patients studied during the project. This 30-day readmission rate reduction is similar to other studies where timely postoperative visits will identify and treat complications such as non-healing wounds and graft thrombosis [6,9,20,21]. Two patients had planned wound revision after virtual visits during the study. Thus, as recommended in other studies, virtual or digital images are suitable for identifying wound care issues [6,9,11,20]. Like other studies, Scholarly Practice Project (SPP) analysis revealed that with return demonstrations and teach-back methods technologically inexperienced older adults participate well in safe and cost-effective virtual care [6,12,20,24]. The virtual postoperative visit rate increased due to the acceptance and adherence of coworkers and patients to the modified discharge process. Staff complaints about virtual visit scheduling declined after project initiation. The clinic had a 15% reduction in postoperative schedule-related messages and no-shows. Decreased elective surgeries and unavailability of providers resulted in low virtual postoperative visit rates during the project.

The selected population was distributed unevenly as more than 95% having an urban residence, unrestricted internet access, and cancer disease. All these factors promoted easy access and the need for constant virtual contact with providers. These same factors also limit the direct replication of the project without modification to other surgical specialties. The project analysis did not establish a statistically significant association between patient education and the observed behavioral patterns of accepting virtual care. The insignificant association between patient education and virtual visit rate was due to no control population, a small sample size, a short interval, and the socioeconomic profile of the population. Therefore, a separate study is advisable to evaluate the influence of patient education and the efficacy of virtual visits among cancer patients. Continuing and comparing similar projects in different specialties with a diverse population as a case-control analysis would be beneficial in establishing virtual postoperative care guidelines. Moreover, workers with high morale and financially resilient stakeholders are essential for the replication of this project.

## Conclusions

The project positively influenced several key patient care quality indicators that impact payer reimbursement, hospital ratings, and provider rankings. For instance, the timely scheduling of virtual postoperative visits resulted in decreased clinic no-show rates, reduced 30-day readmission rates, and lowered surgical site infection rates. The modified discharge process, which employed the teach-back method and demonstrations, helped older patients navigate virtual visits effectively before their actual appointments. Additionally, this process introduced cost-effective practices for older adults with limited technological skills, facilitating their use of virtual care.

The project also promoted optimal utilization of healthcare information technology in a rapidly changing clinical environment influenced by the global pandemic. To ensure sustainability of the project goals and interventions, EMR modifications and staff adherence were implemented. The SPP developed and established a care pathway for postoperative visits within four weeks after surgery, eliminating the need for multiple communications between patients, providers, and clinic staff post-discharge. Virtual postoperative visits increased clinic revenue by replacing traditional postoperative visit slots with new patient visit slots. Many cancer patients, who often have multiple comorbidities, appreciated the convenience and economic benefits of virtual visits.

This project is part of the department’s quality initiative, supported by the administration, and focused on the discharge process for cancer patients undergoing vascular surgery. Multiple studies have shown that postoperative care can be improved using a virtual platform, making the process replicable in other specialties or even during transitions of patient care. Large-scale, multidisciplinary, age-specific discharge modification studies are recommended to address health disparities within the population. Given the extensive positive effects of this project, dissemination of the project outcomes to stakeholders is warranted.
